# Supplementary material for: Language distance and labor market integration of migrants: Gendered perspective
Source: PLoS One. 2024 Apr 18;19(4):e0299936. doi: 10.1371/journal.pone.0299936 (PMC11025911; doi:10.1371/journal.pone.0299936)
Supplement: S1 File — (DOCX) [file pone.0299936.s001.docx]

**Appendices**

Appendix 1: Descriptive statistics of the sample

|  | Men | Women |
| --- | --- | --- |
| N | 2,139 | 2,704 |
| % Labor force participation | 0.823 | 0.697 |
| % Worked last week | 0.692 | 0.551 |
| Weekly working hours | 40.542 | 33.895 |
|  | (11.091) | (12.278) |
| ISEI score | 42.255 | 39.450 |
|  | (16.866) | (18.897) |
| Highest level education |  |  |
| % Low | 0.648 | 0.593 |
| % BA | 0.197 | 0.261 |
| % MA+ | 0.155 | 0.146 |
| Literacy score | 240.322 | 240.320 |
|  | (59.155) | (58.008) |
| % Foreign education | 0.525 | 0.567 |
| Age | 41.351 | 40.853 |
|  | (13.149) | (12.451) |
| % Having children | 0.722 | 0.775 |
| % Living with spouse or partner | 0.722 | 0.775 |
|  |  |  |
| % More than 10 years since migration | 0.682 | 0.653 |
| Linguistics distance | 0.671 | 0.692 |
|  | (0.238) | (0.224) |
| Cultural distance | 2.028 | 1.931 |
|  | (1.028) | (1.025) |
| **Country** |  |  |
| % BEL | 0.034 | 0.033 |
| % CYP | 0.024 | 0.052 |
| % CZE | 0.016 | 0.022 |
| % DNK | 0.223 | 0.214 |
| % ESP | 0.070 | 0.059 |
| % FIN | 0.005 | 0.011 |
| % FRA | 0.101 | 0.083 |
| % GBR | 0.076 | 0.088 |
| % GRC | 0.008 | 0.035 |
| % ISR | 0.165 | 0.162 |
| % KAZ | 0.014 | 0.015 |
| % LTU | 0.012 | 0.014 |
| % NDL | 0.048 | 0.054 |
| % NOR | 0.101 | 0.077 |
| % SVK | 0.011 | 0.011 |
| % SVN | 0.093 | 0.071 |

Appendix 2: Literacy competence of migrants by linguistics distance

|  | (1) | (2) | (3) | (4) | (5) | (6) |
| --- | --- | --- | --- | --- | --- | --- |
| VARIABLES | All | All | Women | Men | Women | Men |
|  |  |  |  |  |  |  |
| Linguistics distance | -88.339*** | -88.598*** | -81.194*** | -92.102*** | -30.023*** | -74.464*** |
|  | (5.513) | (6.937) | (7.545) | (8.144) | (9.745) | (11.597) |
| Female | -4.837*** | -5.216 |  |  |  |  |
|  | (1.486) | (6.354) |  |  |  |  |
| Female *Linguistics distance |  | 0.520 |  |  |  |  |
|  |  | (8.464) |  |  |  |  |
| BA | 38.005*** | 38.009*** | 43.116*** | 31.024*** | 43.771*** | 29.333*** |
|  | (2.105) | (2.105) | (2.675) | (3.385) | (2.916) | (3.829) |
| MA+ | 54.559*** | 54.560*** | 57.453*** | 51.672*** | 57.969*** | 52.849*** |
|  | (2.127) | (2.127) | (2.804) | (3.249) | (2.975) | (3.651) |
| Education in origin country | -2.885* | -2.885* | -2.686 | -3.457 | -1.951 | -5.966** |
|  | (1.666) | (1.666) | (2.214) | (2.525) | (2.435) | (2.906) |
| Age | -1.060*** | -1.060*** | -1.068*** | -1.101*** | -1.190*** | -1.103*** |
|  | (0.081) | (0.081) | (0.108) | (0.123) | (0.118) | (0.140) |
| Having children | 5.120** | 5.123** | 8.108*** | 1.321 | 4.486 | 0.634 |
|  | (2.038) | (2.039) | (2.560) | (3.435) | (2.746) | (3.904) |
| Leaving with a partner | -0.991 | -0.987 | 0.375 | -2.413 | 3.386 | -1.726 |
|  | (1.942) | (1.943) | (2.400) | (3.406) | (2.651) | (3.845) |
| Up to 10 years in the country | 15.592*** | 15.596*** | 19.641*** | 10.281*** | 21.366*** | 8.459** |
|  | (1.926) | (1.927) | (2.581) | (2.917) | (2.808) | (3.362) |
| Cultural distance |  |  |  |  | -13.651*** | -3.599* |
|  |  |  |  |  | (1.705) | (2.008) |
| Constant | 311.415*** | 311.601*** | 299.660*** | 319.007*** | 289.872*** | 313.154*** |
|  | (8.499) | (9.021) | (11.134) | (12.942) | (12.143) | (14.628) |
|  |  |  |  |  |  |  |
| Observations | 4,843 | 4,843 | 2,704 | 2,139 | 2,232 | 1,706 |
| R-squared | 0.250 | 0.250 | 0.270 | 0.240 | 0.295 | 0.241 |

Individual aged 25–65, all models control for include country fixed effect.

Standard errors in parentheses *** p<0.01, ** p<0.05, * p<0.1

Appendix Figure 1:


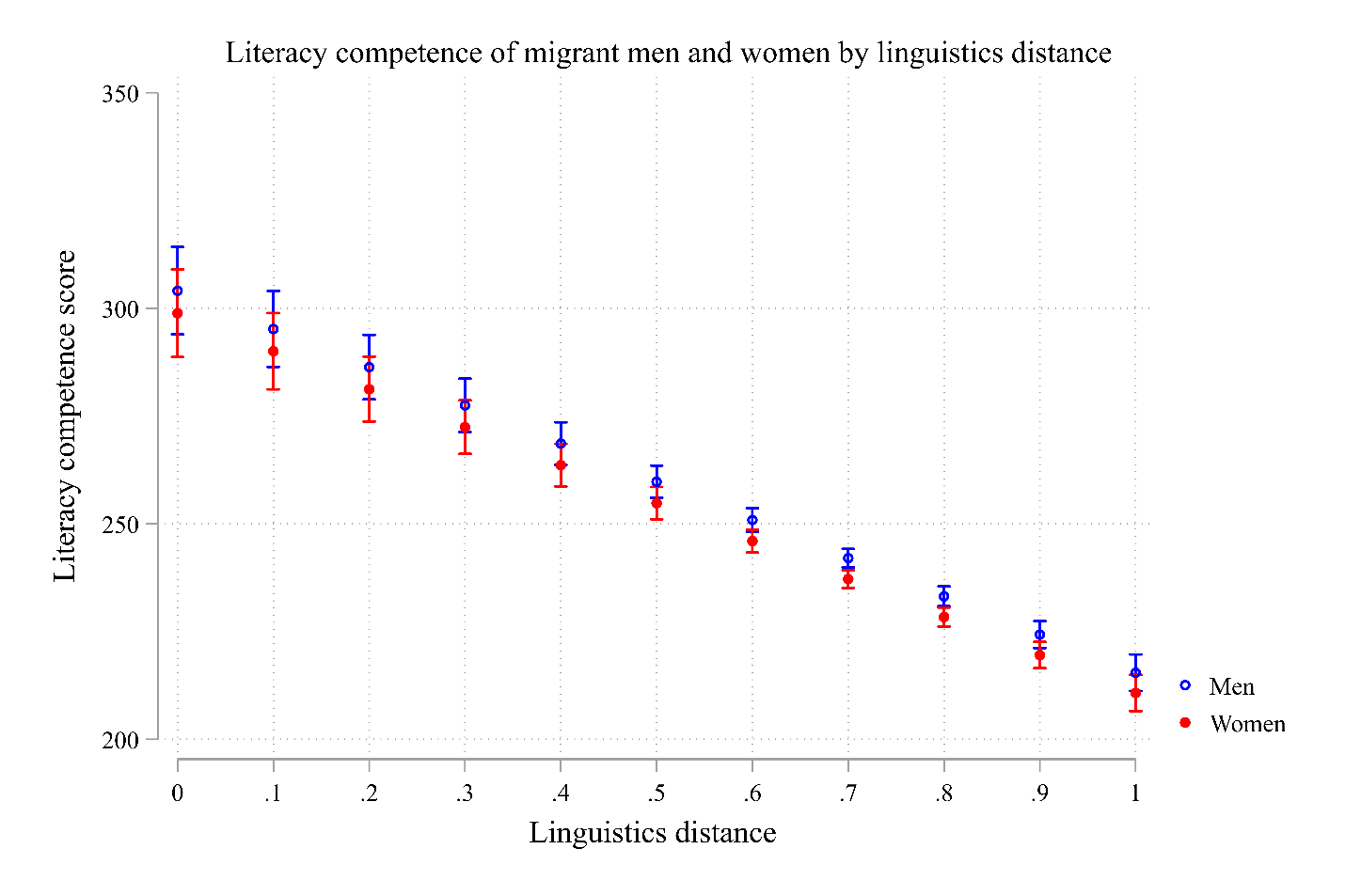


Appendix 3: Labor market outcome of migrants by linguistics differences, cultural distance, and linguistics literacy - **beta coefficients**

|  | (1) | (2) | (3) | (4) | | (1) | (2) | (3) | (4) |
| --- | --- | --- | --- | --- | --- | --- | --- | --- | --- |
|  | LFP | | | | | Employment | | | |
| VARIABLES | Women | Men | Women | Men | | Women | Men | Women | Men |
| Linguistics distance | -0.209*** | 0.051* | -0.201*** | 0.060 | | -0.187*** | -0.043 | -0.172*** | 0.003 |
| Cultural distance |  |  | -0.075*** | -0.028 | |  |  | -0.078*** | -0.081** |
| Literacy competence | 0.124*** | 0.003 | 0.103*** | -0.003 | | 0.135*** | 0.072*** | 0.119*** | 0.073*** |
| BA | 0.076*** | 0.026 | 0.084*** | 0.034 | | 0.103*** | -0.010 | 0.104*** | -0.009 |
| MA+ | 0.077*** | 0.145*** | 0.101*** | 0.160*** | | 0.080*** | 0.148*** | 0.095*** | 0.149*** |
| Education in origin country | -0.011 | 0.097*** | -0.010 | 0.100*** | | 0.001 | 0.094*** | -0.004 | 0.086*** |
| Age | -0.028 | -0.137*** | -0.030 | -0.162*** | | 0.039 | -0.109*** | 0.016 | -0.137*** |
| Having children | 0.040* | -0.131*** | 0.042* | -0.160*** | | 0.066*** | -0.162*** | 0.061*** | -0.186*** |
| Leaving with a partner | 0.022 | -0.095*** | 0.050** | -0.084*** | | 0.013 | -0.133*** | 0.033 | -0.134*** |
| Up to 10 years in the country | 0.006 | -0.022 | 0.017 | -0.043 | | -0.011 | -0.038 | -0.003 | -0.040 |
|  |  |  |  |  | |  |  |  |  |
| Observations | 2,704 | 2,139 | 2,232 | 1,706 | | 2,704 | 2,139 | 2,232 | 1,706 |
| R-squared | 0.092 | 0.068 | 0.107 | 0.079 | | 0.094 | 0.128 | 0.104 | 0.136 |
| VARIABLES | Working hours | | | | | ISEI | | | |
|  | Women | Men | Women | | Men | Women | Men | Women | Men |
| Linguistics distance | -0.077** | -0.076** | -0.101** | | -0.049 | 0.051* | -0.053 |  | 0.060 |
| Cultural distance |  |  | 0.014 | | -0.060 |  |  | -0.080*** | -0.170*** |
| Literacy competence | 0.074*** | 0.183*** | 0.080*** | | 0.204*** | 0.275*** | 0.284*** | 0.272*** | 0.280*** |
| BA | 0.002 | -0.066** | -0.012 | | -0.066** | 0.182*** | 0.201*** | 0.167*** | 0.210*** |
| MA+ | 0.017 | 0.029 | 0.005 | | 0.006 | 0.467*** | 0.399*** | 0.457*** | 0.389*** |
| Education in origin country | -0.013 | 0.042 | 0.005 | | 0.033 | -0.093*** | -0.044* | -0.091*** | -0.077*** |
| Age | 0.092*** | 0.000 | 0.095** | | 0.044 | -0.001 | 0.036 | 0.007 | 0.046 |
| Having children | 0.179*** | -0.023 | 0.201*** | | 0.009 | 0.045* | 0.044 | 0.046* | 0.077** |
| Leaving with a partner | -0.044* | -0.103*** | -0.038 | | -0.123*** | -0.092*** | -0.047* | -0.097*** | -0.068** |
| Up to 10 years in the country | -0.010 | 0.043 | -0.005 | | 0.056 | 0.117*** | 0.095*** | 0.117*** | 0.097*** |
|  |  |  |  | |  |  |  |  |  |
| Observations | 1,624 | 1,544 | 1,341 | | 1,213 | 1,347 | 1,279 | 1,229 | 1,136 |
| R-squared | 0.071 | 0.140 | 0.070 | | 0.152 | 0.464 | 0.343 | 0.461 | 0.372 |

Individuals aged 25–65, all models control for include country fixed effect.

Normalized beta coefficients in parentheses *** p<0.01, ** p<0.05, * p<0.1

Appendix 4: Gender gaps in the labor market outcome of migrants by linguistic proficiency

|  | (3) | (4) | (5) | (6) | (7) | (8) | (9) | (10) |
| --- | --- | --- | --- | --- | --- | --- | --- | --- |
| VARIABLES | LFP | LFP | Employment | Employment | Working hours | Working hours | ISEI | ISEI |
|  |  |  |  |  |  |  |  |  |
| Linguistic proficiency | 0.001*** | -0.000 | 0.001*** | 0.001*** | 0.031*** | 0.038*** | 0.086*** | 0.074*** |
|  | (0.000) | (0.000) | (0.000) | (0.000) | (0.004) | (0.005) | (0.005) | (0.007) |
| Female | -0.181*** | -0.515*** | -0.180*** | -0.345*** | -6.222*** | -2.426 | -5.136*** | -12.007*** |
|  | (0.012) | (0.050) | (0.014) | (0.057) | (0.433) | (1.903) | (0.535) | (2.371) |
| Female# linguistic proficiency |  | 0.001*** |  | 0.001*** |  | -0.015** |  | 0.028*** |
|  |  | (0.000) |  | (0.000) |  | (0.008) |  | (0.010) |
| BA | 0.067*** | 0.060*** | 0.063*** | 0.060*** | -0.989 | -0.927 | 8.219*** | 8.079*** |
|  | (0.018) | (0.018) | (0.020) | (0.020) | (0.615) | (0.616) | (0.744) | (0.744) |
| MA+ | 0.109*** | 0.107*** | 0.127*** | 0.126*** | 0.647 | 0.647 | 17.932*** | 17.918*** |
|  | (0.018) | (0.018) | (0.021) | (0.021) | (0.606) | (0.605) | (0.764) | (0.763) |
| Education in origin country | 0.024* | 0.024* | 0.039** | 0.039** | 0.280 | 0.273 | -2.401*** | -2.408*** |
|  | (0.014) | (0.014) | (0.015) | (0.015) | (0.481) | (0.481) | (0.591) | (0.591) |
| Age | -0.001* | -0.001** | 0.001 | 0.000 | 0.069*** | 0.070*** | 0.032 | 0.029 |
|  | (0.001) | (0.001) | (0.001) | (0.001) | (0.025) | (0.025) | (0.030) | (0.030) |
| Having children | -0.019 | -0.023 | -0.030 | -0.032* | 2.197*** | 2.260*** | 2.020*** | 1.872** |
|  | (0.017) | (0.017) | (0.019) | (0.019) | (0.579) | (0.579) | (0.728) | (0.729) |
| Leaving with a partner | -0.026* | -0.025 | -0.061*** | -0.060*** | -2.461*** | -2.494*** | -3.586*** | -3.469*** |
|  | (0.016) | (0.016) | (0.018) | (0.018) | (0.568) | (0.568) | (0.719) | (0.719) |
| Constant | 0.662*** | 0.848*** | 0.370*** | 0.462*** | 27.265*** | 25.411*** | 15.007*** | 18.159*** |
|  | (0.054) | (0.060) | (0.061) | (0.068) | (1.922) | (2.124) | (2.335) | (2.561) |
|  |  |  |  |  |  |  |  |  |
| Observations | 4,843 | 4,843 | 4,843 | 4,843 | 3,168 | 3,168 | 2,626 | 2,626 |
| R-squared | 0.077 | 0.086 | 0.096 | 0.098 | 0.129 | 0.130 | 0.400 | 0.402 |

Standard errors in parentheses, *** p<0.01, ** p<0.05, * p<0.1

Appendix 5: Labor force participation of migrants by linguistics differences - with country of origin and country of destination fixed effect

|  | (1) | (2) | (3) | (4) |
| --- | --- | --- | --- | --- |
| VARIABLES | All | All | Women | Men |
|  |  |  |  |  |
| Linguistics distance | -0.335*** | -0.108 | -0.471*** | -0.034 |
|  | (0.081) | (0.090) | (0.112) | (0.112) |
| Female | -0.188*** | 0.120** |  |  |
|  | (0.013) | (0.055) |  |  |
| Female *Linguistics distance |  | -0.427*** |  |  |
|  |  | (0.073) |  |  |
| BA | 0.105*** | 0.101*** | 0.101*** | 0.030 |
|  | (0.019) | (0.019) | (0.027) | (0.027) |
| MA+ | 0.116*** | 0.117*** | 0.085*** | 0.132*** |
|  | (0.021) | (0.021) | (0.029) | (0.029) |
| Literacy competence | 0.001*** | 0.001*** | 0.001*** | 0.000 |
|  | (0.000) | (0.000) | (0.000) | (0.000) |
| Education in origin country | 0.016 | 0.017 | 0.004 | 0.072*** |
|  | (0.015) | (0.015) | (0.022) | (0.020) |
| Age | -0.002** | -0.002** | -0.002* | -0.003*** |
|  | (0.001) | (0.001) | (0.001) | (0.001) |
| Having children | -0.030* | -0.033* | 0.012 | -0.133*** |
|  | (0.018) | (0.018) | (0.024) | (0.026) |
| Leaving with a partner | -0.022 | -0.026 | 0.010 | -0.043* |
|  | (0.017) | (0.017) | (0.023) | (0.026) |
| Up to 10 years in the country | 0.018 | 0.016 | 0.029 | -0.036 |
|  | (0.018) | (0.017) | (0.025) | (0.023) |
| Constant | 0.933*** | 0.772*** | 0.764*** | 0.877*** |
|  | (0.121) | (0.123) | (0.186) | (0.150) |
|  |  |  |  |  |
| Country of origin FE | YES | YES | YES | YES |
| Country of destination FE | YES | YES | YES | YES |
|  |  |  |  |  |
| Observations | 4,232 | 4,232 | 2,386 | 1,846 |
| R-squared | 0.179 | 0.186 | 0.252 | 0.241 |

Standard errors in parentheses, *** p<0.01, ** p<0.05, * p<0.1

Appendix 6: Employment of migrants by linguistics differences - with country of origin and country of destination fixed effect

|  | (1) | (2) | (3) | (4) |
| --- | --- | --- | --- | --- |
| VARIABLES | All | All | Women | Men |
|  |  |  |  |  |
| Linguistics distance | -0.314*** | -0.091 | -0.513*** | 0.025 |
|  | (0.091) | (0.101) | (0.119) | (0.139) |
| Female | -0.192*** | 0.111* |  |  |
|  | (0.015) | (0.061) |  |  |
| Female *Linguistics distance |  | -0.419*** |  |  |
|  |  | (0.082) |  |  |
| BA | 0.100*** | 0.096*** | 0.147*** | -0.012 |
|  | (0.022) | (0.022) | (0.029) | (0.034) |
| MA+ | 0.146*** | 0.147*** | 0.110*** | 0.169*** |
|  | (0.023) | (0.023) | (0.031) | (0.036) |
| Literacy competence | 0.001*** | 0.001*** | 0.001*** | 0.001*** |
|  | (0.000) | (0.000) | (0.000) | (0.000) |
| Education in origin country | 0.033** | 0.034** | 0.018 | 0.077*** |
|  | (0.017) | (0.017) | (0.023) | (0.024) |
| Age | -0.001 | -0.001 | -0.000 | -0.004*** |
|  | (0.001) | (0.001) | (0.001) | (0.001) |
| Having children | -0.059*** | -0.062*** | 0.020 | -0.194*** |
|  | (0.020) | (0.020) | (0.026) | (0.032) |
| Leaving with a partner | -0.052*** | -0.056*** | -0.007 | -0.121*** |
|  | (0.019) | (0.019) | (0.024) | (0.032) |
| Up to 10 years in the country | 0.032* | 0.031 | 0.035 | -0.019 |
|  | (0.020) | (0.020) | (0.026) | (0.029) |
| Constant | 0.697*** | 0.539*** | 0.633*** | 0.648*** |
|  | (0.135) | (0.139) | (0.198) | (0.186) |
|  |  |  |  |  |
| Country of origin FE | YES | YES | YES | YES |
| Country of destination FE | YES | YES | YES | YES |
|  |  |  |  |  |
| R-squared | 0.197 | 0.202 | 0.249 | 0.277 |

Standard errors in parentheses, *** p<0.01, ** p<0.05, * p<0.1

Appendix 7: Working hours of migrants by linguistics differences - with country of origin and country of destination fixed effect

|  | (1) | (2) | (3) | (4) |
| --- | --- | --- | --- | --- |
| VARIABLES | All | All | Women | Men |
|  |  |  |  |  |
| Linguistics distance | -10.439*** | -11.039*** | -12.334*** | -3.531 |
|  | (3.022) | (3.292) | (4.225) | (4.384) |
| Female | -6.886*** | -7.750*** |  |  |
|  | (0.489) | (1.939) |  |  |
| Female *Linguistics distance |  | 1.211 |  |  |
|  |  | (2.633) |  |  |
| BA | -1.225* | -1.222* | -0.737 | -2.060** |
|  | (0.705) | (0.706) | (0.968) | (1.037) |
| MA+ | 0.054 | 0.050 | -1.875* | 1.786* |
|  | (0.713) | (0.713) | (1.022) | (1.005) |
| Literacy competence | 0.038*** | 0.038*** | 0.032*** | 0.055*** |
|  | (0.005) | (0.005) | (0.007) | (0.007) |
| Education in origin country | 0.423 | 0.420 | -0.724 | 1.243* |
|  | (0.549) | (0.549) | (0.825) | (0.742) |
| Age | 0.067** | 0.067** | 0.183*** | -0.003 |
|  | (0.029) | (0.029) | (0.044) | (0.037) |
| Having children | 2.255*** | 2.265*** | 6.256*** | -0.748 |
|  | (0.641) | (0.642) | (0.894) | (0.953) |
| Leaving with a partner | -3.005*** | -2.992*** | -1.815** | -4.613*** |
|  | (0.630) | (0.631) | (0.847) | (0.998) |
| Up to 10 years in the country | 1.031 | 1.044 | -0.422 | 1.775** |
|  | (0.664) | (0.664) | (0.998) | (0.890) |
| Constant | 38.143*** | 38.562*** | 25.848*** | 32.292*** |
|  | (4.398) | (4.492) | (7.222) | (5.552) |
|  |  |  |  |  |
| Country of origin FE | YES | YES | YES | YES |
| Country of destination FE | YES | YES | YES | YES |
|  |  |  |  |  |
| Observations | 2,709 | 2,709 | 1,409 | 1,300 |
| R-squared | 0.236 | 0.236 | 0.247 | 0.348 |

Standard errors in parentheses, *** p<0.01, ** p<0.05, * p<0.1

Appendix 8: Occupational prestige of migrants by linguistics differences - with country of origin and country of destination fixed effect

|  | (1) | (2) | (3) | (4) |
| --- | --- | --- | --- | --- |
| VARIABLES | All | All | Women | Men |
|  |  |  |  |  |
| Linguistics distance | 14.401*** | 9.776*** | 9.476* | 13.140** |
|  | (3.460) | (3.706) | (4.889) | (5.205) |
| Female | -5.725*** | -12.662*** |  |  |
|  | (0.560) | (2.099) |  |  |
| Female *Linguistics distance |  | 9.862*** |  |  |
|  |  | (2.877) |  |  |
| BA | 8.043*** | 8.038*** | 6.952*** | 10.711*** |
|  | (0.803) | (0.801) | (1.105) | (1.251) |
| MA+ | 18.134*** | 18.052*** | 19.113*** | 17.675*** |
|  | (0.849) | (0.847) | (1.229) | (1.218) |
| Literacy competence | 0.077*** | 0.077*** | 0.078*** | 0.076*** |
|  | (0.006) | (0.006) | (0.008) | (0.008) |
| Education in origin country | -2.153*** | -2.163*** | -2.907*** | -1.465 |
|  | (0.627) | (0.626) | (0.940) | (0.891) |
| Age | 0.001 | 0.002 | -0.056 | 0.018 |
|  | (0.032) | (0.032) | (0.048) | (0.045) |
| Having children | 1.897** | 2.028*** | 1.850* | 1.133 |
|  | (0.763) | (0.762) | (1.096) | (1.155) |
| Leaving with a partner | -3.225*** | -3.168*** | -3.696*** | -1.083 |
|  | (0.740) | (0.739) | (0.964) | (1.257) |
| Up to 10 years in the country | 4.072*** | 4.199*** | 4.964*** | 4.514*** |
|  | (0.745) | (0.744) | (1.124) | (1.047) |
| Constant | 5.911 | 9.224* | 15.439* | 3.730 |
|  | (4.898) | (4.982) | (7.991) | (6.537) |
|  |  |  |  |  |
| Country of origin FE | YES | YES | YES | YES |
| Country of destination FE | YES | YES | YES | YES |
|  |  |  |  |  |
| Observations | 2,512 | 2,512 | 1,293 | 1,219 |
| R-squared | 0.485 | 0.487 | 0.553 | 0.506 |

Standard errors in parentheses, *** p<0.01, ** p<0.05
